# Clinical Experience With the Nontyphoidal Salmonella Vascular Infection (NTSVI) Score in a Resource-Limited Malaysian Tertiary Center

**DOI:** 10.1155/crdi/9966681

**Published:** 2025-11-14

**Authors:** Weixiang Koh, Nurulyumni Faraha binti Md Sharizam, Seng Hong Koh, Wei Xuan Tuang, Edmund Liang Chai Ong

**Affiliations:** ^1^Department of Internal Medicine, Hospital Sultanah Aminah, Johor Bahru, Johor, Malaysia; ^2^Department of Pathology, Hospital Sultanah Aminah, Johor Bahru, Johor, Malaysia; ^3^Department of Radiology, Hospital Sultanah Aminah, Johor Bahru, Johor, Malaysia; ^4^Department of Medicine, Newcastle University Medicine Malaysia, Iskandar Puteri, Johor, Malaysia

**Keywords:** Malaysia, nontyphoidal Salmonella, resource-limited settings, risk scoring system, vascular infection

## Abstract

Nontyphoidal Salmonella (NTS) bacteremia is an uncommon but potentially fatal infection that predisposes patients to vascular complications, including infectious aortitis. The Nontyphoidal Salmonella Vascular Infection (NTSVI) Score was developed to estimate this risk, with scores ≥ 1 indicating high risk. We describe the first clinical application of the NTSVI Score in a resource-limited Malaysian tertiary care center. Five patients with confirmed NTS bacteremia were assessed using the score and subsequently underwent imaging to evaluate for vascular involvement. Four patients were classified as high risk (scores 3, 2, 1, and 1); two had radiological evidence of infectious aortitis, while two showed no vascular changes. The remaining patient, classified as low risk (score 0), also demonstrated no vascular abnormalities. In this series, the NTSVI Score showed excellent negative predictive value, supporting its potential role as a rule-out tool to avoid unnecessary imaging in low-risk patients, particularly in resource-constrained settings. Although the positive predictive value was modest, the score appeared useful in early risk stratification and guiding further investigation. While limited by small sample size and lack of contemporary validation, this report highlights the potential clinical utility of the NTSVI Score in managing NTS vascular infections in resource-limited settings. To our knowledge, this represents the first reported application of the NTSVI Score in Malaysia, underscoring the need for further evaluation in Southeast Asian populations.

**Summary**

• Nontyphoidal Salmonella bacteremia remains an important cause of morbidity and mortality worldwide due to its risk of vascular involvement.
• The Nontyphoidal Salmonella Vascular Infection (NTSVI) Score offers a simple approach to early risk stratification, supporting timely decisions and conserving resources.
• In our Malaysian tertiary care experience, the NTSVI Score showed a negative predictive value, reinforcing its role as a potential rule-out tool for vascular infection.
• This is the first reported application of the NTSVI Score in Malaysia, highlighting its possible utility in resource-limited Southeast Asian settings.

## 1. Introduction

Nontyphoidal Salmonella (NTS) bacteremia is a clinically important infection due to its potential to cause invasive vascular complications, particularly infectious aortitis. Early recognition of these infections is essential, as timely initiation of prolonged antimicrobial therapy combined with surgical intervention can significantly improve survival outcomes [1, 2]. However, diagnosing vascular involvement remains challenging, especially in resource-limited settings where access to advanced imaging is restricted.

The Nontyphoidal Salmonella Vascular Infection (NTSVI) Score was developed in Taiwan as a simple clinical tool to estimate the risk of vascular involvement in patients with NTS bacteremia. It incorporates both positive predictors (male sex, hypertension, coronary artery disease, and serogroup C1 infection) and negative predictors (malignancy and immunocompromised status), yielding a range from −2 to +4. A cutoff of ≥ 1 has been associated with high sensitivity (95%) but modest specificity (45.3%) [3].

Despite these promising findings, the NTSVI Score has not been validated outside its original setting. In regions such as Southeast Asia, where NTS bacteremia remains clinically relevant and access to advanced imaging is often limited, the score may be most useful as a rule-out tool to avoid unnecessary investigations. This case series presents the first application of the NTSVI Score in a Malaysian tertiary care center, exploring its potential utility in early risk stratification and clinical decision-making in resource-limited settings.

## 2. Case Presentation

Five patients with confirmed NTS bacteremia admitted to a Malaysian tertiary care center in 2024 were reviewed. Diagnosis was established through positive blood cultures processed using standard microbiological techniques (Table 1).

The NTSVI Score was calculated for each patient and stratified as high risk (≥ 1) or low risk (< 1). All patients underwent vascular imaging to evaluate for vascular involvement, including infectious aortitis.

Concordance between NTSVI classification and imaging findings was assessed descriptively on a case-by-case basis.

### 2.1. Case 1

A 62-year-old Chinese man with ischemic heart disease, heart failure with reduced ejection fraction, and hypertension presented with acute breathlessness, bilateral leg swelling, fever, loose stools, and interscapular pain. He had a 30-pack-year smoking history but no other significant risk factors. On admission, he was managed for acute decompensated heart failure and presumed infective gastroenteritis (Table 2).

**Figure 1:**
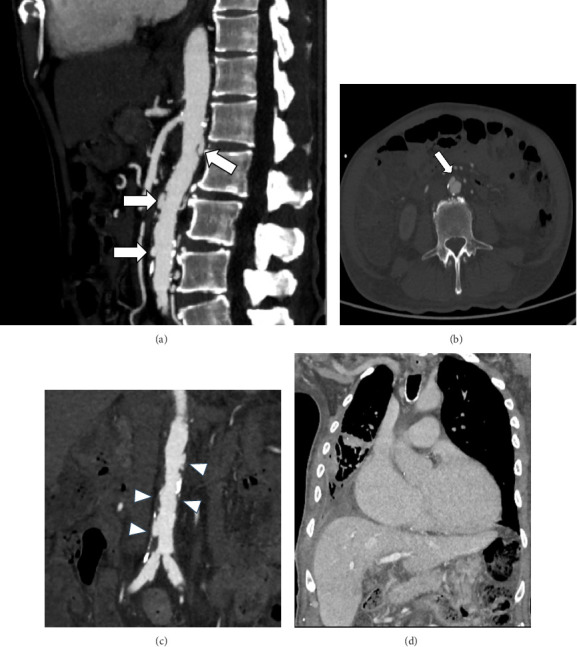
(a) CTA abdominal aorta in sagittal view shows multiple small aneurysms (white arrows). (b) CTA abdominal aorta in axial view shows multiple small aneurysms (white arrows). (c) CTA abdominal aorta in coronal view demonstrates features of aortitis involving the abdominal aorta and bilateral common iliac arteries (white arrows). (d) CECT thorax in coronal view shows cardiomegaly with consolidation of the right lower lobe suggestive of pneumonia.

Blood cultures yielded NTS, and targeted antimicrobial therapy was initiated. His NTSVI score was +3 (male sex, hypertension, and coronary artery disease), indicating high risk for vascular infection. Contrast-enhanced CT angiography revealed multiple focal outpouchings of the abdominal aorta and bilateral proximal common iliac arteries, consistent with infectious aortitis; the largest lesion measured 0.5 × 1.0 × 1.2 cm (AP × W × CC) (Figures 1(a), 1(b), 1(c), and 1(d)).

He was deemed unfit for surgical intervention due to comorbidities and was managed conservatively with intravenous antibiotics. He was discharged in stable condition and remained well at 2-month follow-up. Retrospective serotyping identified *Salmonella enteritidis* (serogroup D). Unfortunately, he passed away 3 months later, likely due to progression of heart failure and underlying comorbidities.

### 2.2. Case 2

An 80-year-old Malay woman with a background of hypertension and ischemic heart disease presented with a 10-day history of fever, loose stools, and abdominal pain. She had no other significant past medical history. She was clinically dehydrated with markedly elevated inflammatory markers (Table 2). She was initially managed for intra-abdominal sepsis.

Blood cultures subsequently yielded NTS species, and antimicrobial therapy was tailored according to sensitivity results. Her NTSVI Score was +2 (hypertension and ischemic heart disease), categorizing her as high risk for vascular involvement. Contrast-enhanced CT angiography revealed multiple foci of infectious aortitis involving both the thoracic and abdominal aorta.

Due to advanced age and comorbidities, the patient and her family declined surgical intervention. Despite intravenous antibiotic therapy, her condition progressively deteriorated. She was discharged against medical advice with an end-of-life care package arranged at home and, unfortunately, passed away within 1 week of discharge. Retrospective serotyping identified the isolate as *Salmonella enteritidis* (serogroup D).

### 2.3. Case 3

A 69-year-old Chinese woman presented to the emergency department with a four-day history of fever, loose stools, vomiting, and reduced appetite. Her past medical history was notable for end-stage kidney disease on regular hemodialysis, diabetes mellitus, and ischemic heart disease, for which she had undergone coronary stenting twice. She reported occasional consumption of raw food, with the last exposure occurring approximately 2 weeks before presentation.

On admission, she was diagnosed with infective acute gastroenteritis complicated by sepsis (Table 2) and commenced on empiric antibiotics. Both stool and blood cultures subsequently yielded NTS, later identified as *Salmonella enteritidis* (serogroup D). Her NTSVI score was calculated at +1 (hypertension and ischemic heart disease, offset by −1 for immunocompromised state due to end-stage renal disease).

Contrast-enhanced CT angiography demonstrated normal thoracic and abdominal aortic dimensions, with no evidence of vascular involvement. Clinically, her condition improved with intravenous antibiotics, although she developed persistent diarrhea during hospitalization. Further testing confirmed Clostridium difficile infection, for which she completed a 10-day course of oral vancomycin. She was discharged in stable condition following completion of 2 weeks of intravenous antibiotics.

At 6-week follow-up, the patient remained well with complete resolution of her gastrointestinal symptoms.

### 2.4. Case 4

A 52-year-old Malay man with a history of psoriasis on methotrexate presented with fever, chills, and rigor. His past medical history was notable for prior opportunistic infections, including smear-negative pulmonary tuberculosis and cytomegalovirus pneumonitis, both of which had been treated. He denied high-risk behaviors but reported recent participation in a pool party at Taiwan and habitual consumption of half-boiled eggs.

On admission, he was managed for Gram-negative sepsis, with laboratory investigations showing elevated inflammatory markers and thrombocytopenia (Table 2). Blood cultures subsequently grew NTS, with serotyping pending at the time. His initial NTSVI Score was presumed +1, with positive points attributed to male sex and presumed serogroup C1 infection, taking into consideration his recent travel history to Taiwan and a negative point for his immunocompromised status due to methotrexate therapy.

Contrast-enhanced CT of the thorax, abdomen, and pelvis demonstrated no evidence of vascular infection or intra-abdominal collection. Subsequent serotyping confirmed *Salmonella enteritidis* (serogroup D), leading to a revised NTSVI score of 0, categorizing him as low risk. Given his immunocompromised status, he was treated with a 6-week course of outpatient parenteral antibiotics, which he completed successfully while remaining clinically well throughout.

### 2.5. Case 5

A 77-year-old Chinese lady presented with a three-day history of epigastric pain, shortness of breath, bilateral leg swelling, and productive cough with clear sputum. She had recently been discharged after treatment for rapid atrial fibrillation (AF) with decompensated heart failure and coagulopathy due to warfarin overdose. Her past medical history included diabetes mellitus, hypertension, and congestive heart failure with AF. She had left breast malignancy treated with left breast mastectomy, radiotherapy, and chemotherapy 3 years prior.

She was treated for fast atrial fibrillation which resulted in decompensated cardiac failure on admission, complicated with acute dyspepsia and hospital acquired pneumonia (Table 2). Empiric antibiotics were initiated, and blood cultures subsequently yielded NTS species. An abdominal ultrasound demonstrated a normal aortic diameter with no evidence of aneurysm. Her calculated NTSVI Score was +1 (hypertension). According to the original NTSVI definition, malignancy refers to an active solid tumor or hematologic disease requiring chemotherapy. As her breast cancer was in remission, it was not considered active and therefore did not contribute a negative point.

Persistent epigastric pain prompted contrast-enhanced CT of the thorax and abdomen, which revealed findings consistent with cholecystitis with cholelithiasis. The surgical team was consulted for management of symptomatic cholelithiasis, with a plan for elective cholecystectomy. She completed a 10-day course of intravenous antibiotics and was discharged in stable condition. Retrospective serotyping identified the isolate as *Salmonella enteritidis* (serogroup D).

## 3. Discussion

Our series represents the first report of NTSVI Score application in Malaysia, highlighting its potential clinical value for early risk stratification of NTS vascular infections. Patients were categorized into high-risk (NTSVI score ≥ 1) and low-risk groups according to the original scoring algorithm [3].

The scoring system was applied consistently, following the original definitions outlined by Chen et al. [3]. In this model, malignancy refers specifically to an active solid tumor or hematologic disease requiring chemotherapy. Accordingly, a history of malignancy in remission was not assigned a negative point, as illustrated in Case 5, where the patient's breast cancer had been in remission for 3 years. This distinction ensured that only active disease states influenced risk estimation.

Overall, the NTSVI Score demonstrated excellent negative predictive value, reliably identifying patients at low risk for vascular infection and potentially reducing the need for unnecessary imaging. In contrast, the positive predictive value was modest, reflecting the small sample size and the multifactorial nature of infectious aortitis.

Among the five cases, two high-risk patients had radiologically confirmed infectious aortitis. Open surgical repair remains the standard of care for infectious aortitis, although endovascular therapy may be considered in selected cases [2, 4–6]. Both of our affected patients in this series were clinically unfit for surgery; nevertheless, early presentation facilitated timely identification and initiation of targeted therapy, which likely contributed to transient improved outcomes. Unfortunately, both patients subsequently succumbed postdischarge, underscoring the importance of incorporating patient-specific factors such as age, cardiovascular comorbidities, and frailty into clinical decision-making [7].

Advanced age is a well-recognized risk factor for invasive Salmonella infection and vascular involvement, largely due to age-related endothelial injury, atherosclerosis, and impaired immune responses. All patients in our series were older than 50 years, aligning with prior studies in which older age correlated strongly with both bacteremia and vascular complications [3, 7]. The predominance of elderly patients in this series may, therefore, reflect an inherent predisposition to vascular infection, even among those categorized as low risk by NTSVI criteria. Incorporating age as a weighted variable may, therefore, enhance predictive performance in aging populations.

In contrast, three patients with NTSVI Score of 1 had no radiological evidence of vascular involvement, including one with concurrent Clostridium difficile infection. Another patient initially presumed to have serogroup C1 infection was recategorized as low risk (NTSVI Score 0) after serotyping confirmed serogroup D. These findings support the NTSVI Score potential as a pragmatic tool to rule out vascular infection and avoid unnecessary imaging.

CTA aorta remains the gold standard for evaluating thoracic and abdominal vascular lesions [8]. In resource-limited tertiary care settings, judicious imaging is essential to optimize diagnostic yield, ensure patient safety, and conserve healthcare resources. Implementation of a validated risk stratification protocol such as the NTSVI Score may help prioritize imaging for high-risk patients while reducing unnecessary exposure to radiation and contrast.

It is noteworthy that the NTSVI Score was derived from a Taiwanese cohort, where serogroup C1 was the predominant isolate at the time, whereas serogroup D is more prevalent in Malaysia. Despite this epidemiological difference, the score maintained excellent negative predictive value, reinforcing its utility as a screening tool in different population. Future recalibration of the score to reflect local serotype distribution and demographic factors, including age, may further enhance its clinical accuracy.

A key limitation of the score's application lies in the delay of serotype identification, which typically requires up to 2 weeks. As a result, initial NTSVI scoring was performed without real-time serogroup information. Nonetheless, this limitation has limited clinical impact, as patients with scores ≥ 1 based on other variables remain classified as high risk for vascular infection regardless of serotype. Future studies should aim to validate and refine the NTSVI Score in larger Southeast Asian cohorts, incorporating contemporary microbiological trends, serotype patterns, and regional disease characteristics.

## 4. Conclusion

The NTSVI Score shows promise as a practical and clinically relevant tool for early risk stratification of NTS vascular infections, particularly in resource-limited tertiary care settings. In our series, the score effectively distinguished low-risk patients, potentially reducing unnecessary imaging and focusing attention on those at higher risk for timely intervention. The difference in serotype distribution, predominantly C1 in Taiwan at the time of score development versus D in Malaysia, underscores the need to consider regional epidemiological variations and suggests that local adaptation or recalibration of the NTSVI Score could improve its predictive accuracy. While limited by sample size and retrospective application, our findings underscore the potential utility of the NTSVI score as a rule-out tool and as a guide for optimizing clinical management. Further prospective studies across larger and more diverse Southeast Asian cohorts are warranted to validate its accuracy, refine its applicability, and assess its impact on patient outcomes.

## Figures and Tables

**Table 1:** Summary of cases.

Case	Gender	Age (year)	Comorbid	Symptoms	Onset till presentation (day)	NTSVI score^∗^	Presentation till CTA (day)	Vascular infection	Treatment	*Salmonella* serotype (serogroup)	Outcome
1	Male	62	HTN HFrEF IHD	Breathlessness Bilateral leg swelling Loose stool Fever Interscapular pain	2	3	3	Yes	Medical Not fit for surgical intervention	*Salmonella enteritidis* (D)	Death
2	Female	80	HTN IHD	Fever Loose stool Abdominal pain	10	2	8	Yes	Medical Not fit for surgical intervention	*Salmonella enteritidis* (D)	Death
3	Female	69	ESKD DM HTN IHD	Fever Loose stool Vomiting Reduced appetite	4	1	2	No	Medical	*Salmonella enteritidis* (D)	Discharged
4	Male	52	Psoriasis (DMARD)	Fever Chills and rigor	3	0	3	No	Medical	*Salmonella enteritidis* (D)	Discharged
5	Female	77	DM HTN CCF AF Breast cancer	Breathlessness Epigastric pain Cough Orthopnea Bilateral leg swelling	3	1	10	No	Medical	*Salmonella enteritidis* (D)	Discharged

*Note:* HTN, hypertension.

Abbreviations: AF, atrial fibrillation; CCF, congestive cardiac failure; CTA, computed tomography aortography; DM, diabetes mellitus; DMARD, disease modifying antirheumatic drugs; ESKD, end stage kidney disease; HFrEF, heart failure reduced ejection fraction; IHD, ischemic heart disease; NTSVI, Nontyphoidal Salmonella Vascular Infection.

^∗^NTSVI score ≥ 1 indicates high risk for vascular infection in NTS bacteremia patients and warrants imaging 1.

**Table 2:** Summary of vital signs and blood investigations upon presentation.

Case		1	2	3	4	5
Blood pressure (mmHg)	Systolic 120 Diastolic 80	141/79	152/83	106/58	151/85	143/98
Heart rate (bpm)	60–100	98	102	78	121	110
Temperature (°C)	< 37.5	37.0	38.3	37.6	37.8	36.7
Oxygen saturation (%)	95–100	97	96	97	95	99
Supplemental oxygen		VM 60	RA	RA	RA	VM 40
Hemoglobin (g/L)	Male 11–13 Female 10–12	130	108	101	146	109
White cell count (× 10^9^/L)	4–12	18.3	24.5	18.0	10.9	12.3
Platelet (× 10^9^/L)	150–400	230	285	205	29	270
C-reactive protein (mg/L)	< 5	119.3	230.4	154.7	148.3	58.3

Abbreviations: RA, room air; VM, venturi mask.
